# Detection of SARS-CoV-2 in Wastewater Northeast of Mexico City: Strategy for Monitoring and Prevalence of COVID-19

**DOI:** 10.3390/ijerph18168547

**Published:** 2021-08-13

**Authors:** José Roberto González-Reyes, María de la Luz Hernández-Flores, Jesús Eduardo Paredes-Zarco, Alejandro Téllez-Jurado, Omar Fayad-Meneses, Lamán Carranza-Ramírez

**Affiliations:** 1 Investigación Aplicada para el Bienestar Social y Ambiental Asociación Civil (INABISA A.C.), Pachuca 42088, Mexico; contacto@inabisa.com.mx (J.R.G.-R.); jeparedes@ciencias.unam.mx (J.E.P.-Z.); 2 Consejo Ejecutivo del Complejo Científico y Tecnológico Sincrotrón, San Agustín Tlaxiaca 42163, Mexico; omarfayad@hidalgo.gob.mx (O.F.-M.); lamancarranza@hidalgo.gob.mx (L.C.-R.); 3 Laboratorio de Agrobiotecnología, Universidad Politécnica de Pachuca, Carretera Pachuca-Cd. Sahagún km 20, Zempoala 43830, Mexico; alito@upp.edu.mx

**Keywords:** environmental policy, wastewater-based epidemiology (WBE), wastewater, epidemiological monitoring, prevalence

## Abstract

A month-long wastewater sampling project was conducted along the northeast periphery of Mexico City, specifically in the state of Hidalgo, to assess the presence of SARS-CoV-2. To determine the prevalence of infection and obtain a range of COVID-19 cases in the main metropolitan zones. Viral RNA residues (0–197,655 copies/L) were measured in wastewater from the five central municipalities in the state. By recording the number of RNA viral copies per liter, micro-basins delimitation, demographic and physiological data, an interval of infected people and virus prevalence was estimated using a Monte Carlo model (with 90% confidence) in the micro-basin of five municipalities with metropolitan influence or industrial activity. Our procedure determined that the percentage of the infected population ranges from 1.4% to 41.7%, while the official data reports 0.1–0.3%. This model is proposed as a helpful method of regional epidemiological monitoring through the analysis of viral prevalence.

## 1. Introduction

Since the detection of a new type of SARS-COV-2 virus in the city of Wuhan, China, prevalent studies of different types have been carried out by [1,2]. The use of wastewaters in prevalence was conducted first for SARS-CoV and MERS-CoV [3], since prevalence is a metric that accurately tracks the behavior of a pandemic.

The SARS-CoV-2 virus is the etiological agent of coronavirus disease 2019 (COVID-19). Because the virus uses the angiotensin converting enzyme (ACE2) as a receptor to enter human cells [4] and there is high expression of ACE2 messenger RNA in the gastrointestinal system ([5,6,7]). This evidence supports the possibility of SARS-CoV-2 replication in the gastrointestinal tract [8].

Some clinical studies reported prolonged fecal shedding of SARS-CoV-2 RNA for up to seven weeks after the first onset of symptoms ([6,9]) or other body fluids [10]. Another study reported that viral RNA could be detected in stool in 81.8% of cases [11].

Keeping in mind that there is scientific evidence of the detection of viable SARS-CoV-2 in feces of patients with COVID-19 ([9,12]) and the presence of virus RNA in wastewater ([1,13]), it is necessary to create methods using wastewater as a possible source of information for epidemiological studies and its risks to human health [8]. The presence of SARS-CoV-2 in wastewater samples is already confirmed by studies from different countries ([1,9,13]). To quote an example, “Viral loads of up to 1.9 × 104 copies/L have been detected in wastewater in locations close to departments receiving COVID-19 patients in Wuhan, China [14]”.

The presence of SARS-CoV-2 viral RNA in wastewater makes it possible to utilize it as a surveillance tool for invasion, prevalence, molecular epidemiology, and possible eradication of the virus in a community [8]. For example, ([13]) reported the prevalence level of SARS-CoV-2 by measuring the viral RNA copies quantified by the RT-qPCR technique found from sewage in a basin of Australia. The wastewater surveillance and Monte Carlo data suggested a measure of the prevalence of SARS-CoV-2 infection of 9.6% in the studied basin over six days.

The increasing severity of the pandemic could be related to the lack of adequate testing trials to quickly and accurately identify patients infected with SARS-CoV-2 [15]. In addition, asymptomatic and pre-asymptomatic SARS-CoV-2 infected patients are highly contagious and, given the lack of appropriate screening assays, many SARS-CoV-2 infected patients have had contact with uninfected individuals before being identified for home isolation or hospitalization [16]. Wastewater-based epidemiology of human viruses can be a useful tool for population-scale monitoring of SARS-CoV-2 prevalence and epidemiology to help prevent further spread of the disease, particularly within urban centers [17].

This study aimed to analyze SARS-CoV-2 viral residues in wastewater from 5 municipalities found in metropolitan areas (Pachuca de Soto, Mineral de la Reforma, Tula de Allende, Tizayuca and Tepeapulco) of the great importance of the State of Hidalgo (northeast of Mexico City). Through its detection and quantification using the RT-qPCR technique for modeling the results using the Monte Carlo model to estimate the number of infected people in the study areas and the prevalence of SARS-CoV-2.

## 2. Case Study

The study area was limited to five municipalities located northeast of Mexico City, in the State of Hidalgo, where five central metropolitan municipalities were chosen: Pachuca de Soto, Mineral de la Reforma, Tula de Allende, Tizayuca, and Tepeapulco. These municipalities are home to an essential proportion of the total population of the State of Hidalgo. For practical terms, these five municipalities have been named as follows, respectively: “Municipality in the Main Metropolitan Area of the State of Hidalgo (MMHS)”, “Municipality on the East Side of the Main Metropolitan Area of the State of Hidalgo (MEMHS)”, “Municipality in the West with Predominant Food and Chemistry Industry (MWFC)”, “Municipality in the North of Mexico City Metropolitan Area (MNMC)” and “Municipality in the East with Predominant Metal-Mechanic Industry (MWFC)”.

Data number of infected and the population for the year 2020 and the place taken at the state level are presented in Table 1.

**Figure 1:**
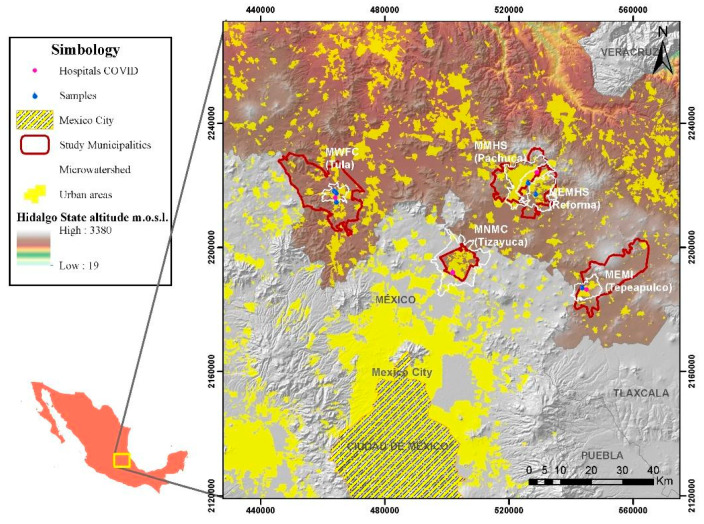
Study area and sampling site of five municipalities of the State of Hidalgo.

In all the municipalities studied, a hospital called “COVID HOSPITAL” consists of existing hospitals with new infrastructure, specifically for COVID-19 patients’ treatment. In most sampling points, the samples contained wastewater coming from any COVID Hospital to guarantee the SARS-CoV-2 viral load in the samples. Figure 1 shows the location of sampling points in the northeast of Mexico City.

## 3. Materials and Methods

### 3.1. Micro-Basin Determination

In Hidalgo, as well as other regions in other developing countries, the mapping of sewage and drainage systems are not complete. Because of this, it was necessary to establish major sampling points in the same sites where the majority of wastewater discharge converges in the chosen municipalities, known as micro-basins. Once these sampling points were determined, we delimited the water runoff in the micro-basins. This defines the maximum and minimum population that discharges its residual waters in a determined micro-basin. This delimitation process was conducted through the hydrology tool from the ARCGIS software. 

### 3.2. Sampling Strategy

Wastewater samples were carried out in volumes of 1 L every 6 h for 24 continuous hours. The samples were stored at a low temperature (4 °C). During the sampling, the flow rate of the sampled sites was calculated. The samplings were carried out in 5 municipalities at similar times with a 10-min difference average until a compound mix of 24 h was obtained, which would allow a comparison of the results. 

MMHS and MEMHS have been operating wastewater treatment plants (WWTP); the rest of the sampling sites were open-air channels. In MWFC 2, one sample from the Tula River was collected, which is fed by the wastewater from Mexico City (the most significant metropolitan area of the Mexican Republic) and is used in agricultural activities within the territory of the State of Hidalgo [19]. MWFC 3 was collected directly from local drainage to 3.5 km of distance north from MWFC 2; its purpose was to estimate the contribution of SARS-CoV-2 from a specific area of the municipality without the sample being diluted in the Tula River.

Another sample is not considered in the Monte Carlo model because it was intended to generate the baseline for viral remains content from municipalities previously to Tula de Allende (MWFC).

The group of samplers used personal protective equipment (PPE), which consisted mainly of N95 face masks, gloves, boots, Tyvek type suits, and masks.

### 3.3. Viral RNA Residues Determination

Once the samples were collected, a 150 mL aliquot, in triplicate, was placed in a water bath at 60 °C for 90 min, as a viral deactivation method. Later, the samples were packed and sent to BioBot laboratories, in Cambridge, MA, USA, for SARS-CoV-2 determination based on the methodology reported by ([20]). The method is described below. The untreated wastewater was filtered through a 0.2 µm membrane (Millipore Sigma, St. Louis, Missouri, United States) to remove bacterial cells and wastes. The filters were discarded, as initial tests revealed little or no viral RNA on the filters. Then 4 g of polyethylene glycol 8000 (8% *w*/*v*, Millipore Sigma) and 0.9 g of NaCl (0.3 M, Millipore Sigma) were added to 40 mL of filtrate and centrifuged at 12,000× *g* for two hours or until a pellet was visible. The viral sediment was resuspended in Trizol (Thermofisher, Waltham, Massachusetts, United States) for RNA extraction, followed by reverse transcription (reverse transcriptase, NEB) and qPCR (TaqMan rapid forward master mix, Thermofisher) with CDC primers (IDT) for the nucleocapsid N gene. The positive control (IDT) is a plasmid containing the complete SARS-CoV-2 nucleocapsid gene and is used to create the standard curves for primers N1, N2, and N3. The result is reported in RNA copy number per liter of wastewater.

The number of RNA viral copies per liter was one of the variables used in the Monte Carlo model to estimate the number of infected people per study area.

One factor that could affect the determination of the concentration of the viral residues was the rainfall presence. The precipitation level in the meteorological stations closest to each of the sampling points is reviewed, and its impact on possible dilution is analyzed.

### 3.4. Number of Infected People and Prevalence through Monte Carlo Model

The prevalence of SARS-CoV-2 infection within the micro-basins was estimated using an equation representing a mass balance of the total number of viral RNA copies each day ([13]), in the municipalities’ wastewater and other variables shown in the following equation.

(1) $$IN=\frac{\left(SARSW\right)\left(WW\right)\left(IMB\right)}{\left(H\right)\left(SARSH\right)}$$

where: *IN*: Estimated number of infected people (people); *SARSW*: number of copies of SARS-CoV-2 R.N.A. per liter of wastewater (copies/L); *WW*: wastewater per capita (L); *IMB*: number of inhabitants in the micro-basin (people); *H*: feces per capita (g); *SARSH*: number of copies of SARS-CoV-2 R.N.A. per gram of stool (copies/g).

The uncertainty in the independent variables was incorporated using a Monte Carlo model run on Oracle Crystal Ball software. The SARS-CoV-2/L RNA copies from wastewater were modeled as point estimates for each detection date and sampling location. The daily flow of wastewater was calculated using the product of the number of inhabitants that dump wastewater into the micro-basin and the average wastewater per capita generated in each municipality according to the Ministry of the Environment and Natural Resources of the State of Hidalgo [21].

The minimum and maximum values of the inhabitants that influence the micro-basin were incorporated through an even distribution curve. Per capita, wastewater production was incorporated into the model through a triangular distribution curve at an interval between 100–200 L/person and 150 L/person as the most probable number [21].

The feces’ daily mass per person per day was modeled as a normal distribution with a mean of 128 g/person/day and a standard deviation of 29 g/person/day [22]. Finally, the shedding rate of SARS-CoV-2 RNA copies/g from feces was modeled as an even distribution from 10,000 copies/g to 1,000,000 copies/g ([13]). The results indicate the number of infected inhabitants in a specific micro-basin of the municipality, in two sampling times per study area. 

The micro-basins represent a territorial percentage of each municipality. Therefore, the Monte Carlo model results represent a part of the population. For this reason, we suppose a homogeneity in infected people distribution to extrapolate our result to municipality level.

We run 10,000 scenarios in each Monte Carlo model, with a 90% confidence interval. Given the significant uncertainties associated with the model, we consider the median like a conservative estimator for infected persons, in part because this value is less sensitive to extreme values drawn from the input distributions ([13]). At the same time, extreme values of the resulting distribution of each model were used as an error margin and are represented between parenthesis in the results section.

Prevalence was calculated through the ratio between the number of people estimated with infection over the total number of inhabitants in the municipality. 

## 4. Results

### 4.1. Micro-Basin Population and General Viral Waste Determination

**Figure 2:**
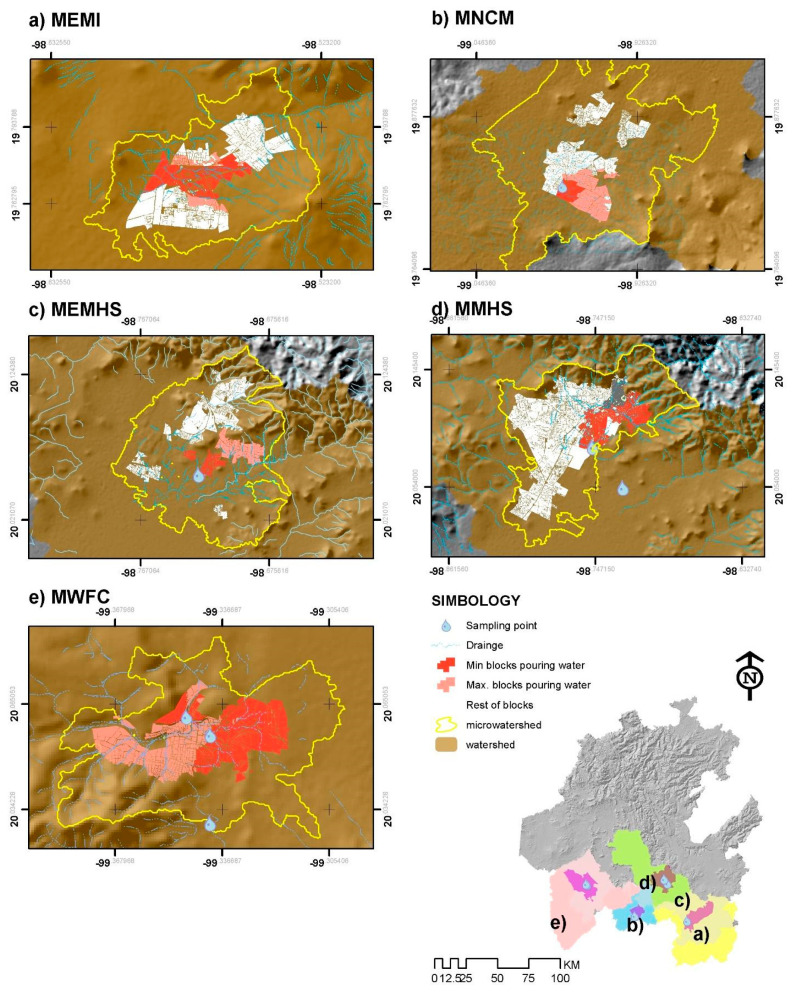
Geographic definition of the micro-basins and sampling sites of five municipalities of the State.

We obtain the next micro-basins where the population generates the wastewater sampling in each studied area. Due to the uncertainty of the urban blocks that generate the wastewater, we delimitate a minimum and maximum block that pour water into each micro basin. Figure 2 shows micro-basin delimitation in each municipality studied, streams, and location of sample sites.

**Figure 3:**
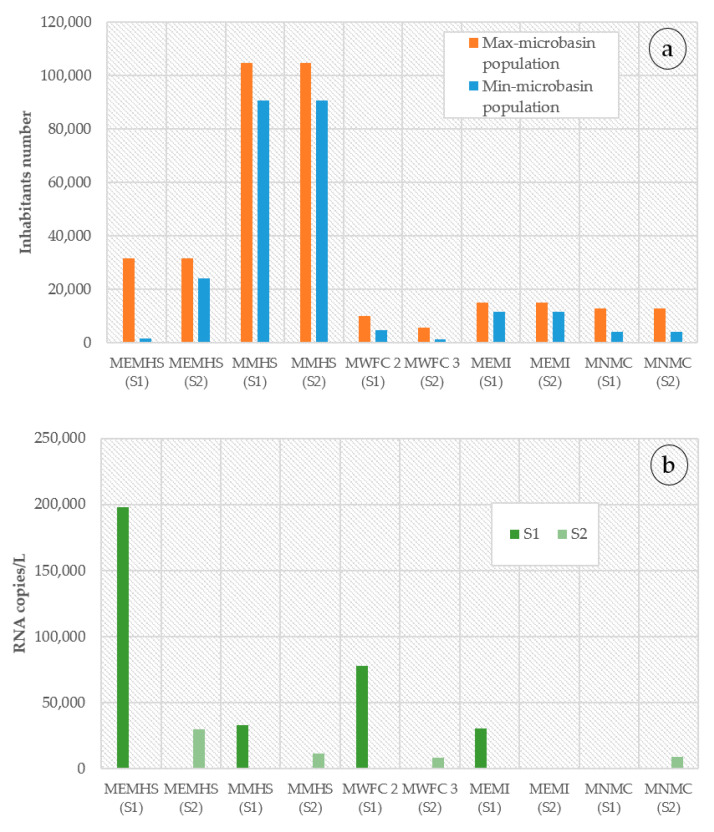
(**a**). The minimum and maximum micro-basin population sizes are indicated in blue and orange, respectively. (**b**). Concentration of SARS-CoV-2 RNA. The concentration of viral RNA (number of RNA copies per liter of wastewater) is indicated with dark green bars (S1 samples) and light green bars (S2 samples).

On average, the number of inhabitants in the micro-basins was: MEMHS (27,839) and MMHS (97,627), MEMI (13,310), followed by MNMC (8556), MWFC 2 (7310), MWFC 3 (3468). The viral RNA residues/L content was of the order of magnitude of 10^3^ and up to 10^5^. A decrease is shown in RNA copies/L during the second sampling for most cases in the next figure. An exception is in MEMI and MNMC, in which first and second samples, respectively, have not had SARS-CoV-2 RNA copies. Figure 3 shows a comparison of maximum and minimum population per micro basin and the RNA copies/L.

For the first sampling work carried out on June 29th, the results indicate that the place that presented the highest number of viral RNA copies was MEMHS with 1.97 × 10^5^ copies/L, secondly, MWFC 2 in Tula de Allende Center, followed by MWFC baseline sample and MMHS, the values were 7.8 × 10^4^, 5.6 × 10^4^, and 3.2 × 10^4^ RNA/L copies, respectively. MEMI had a value of the order of 3.0 × 10^4^ RNA/L copies, while they were not detected viral copies in MNMC.

**Figure 4:**
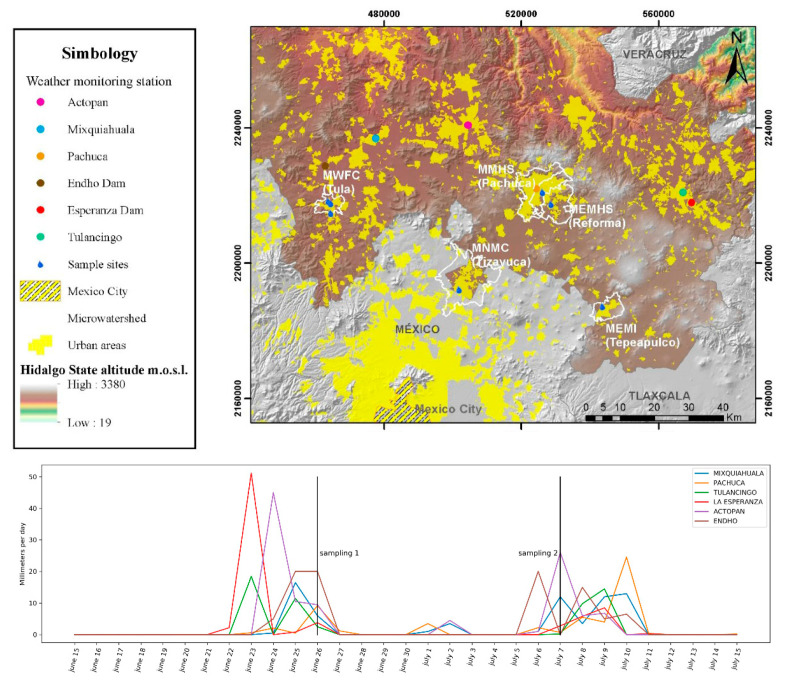
Sampling places and data from weather monitoring stations about precipitation (mm per day).

For the second sampling (July 6th), from the highest to the lowest (RNA viral copies), we obtain the following results: MEMHS (2.98 × 10^4^), MMHS (1.17 × 10^4^), MNMC (9.23 × 10^3^), and MWFC 3 (8.15 × 10^3^). In MEMI and MNMC, RNA viral copies were not detected. Figure 4 shows sampling places, weather monitoring stations, and precipitation measured during sampling days. It is essential to point out that, for the MMHS samples, the low number of viral RNA copies could be related to the type of watercourse since it feeds on wastewater and rainwater from Pachuca de Soto’s upper urban area.

### 4.2. Baseline Comparison in MWFC

MWFC baseline and MWFC 2 correspond to the Tula de Allende River. The first of these samples is behind Tula de Allende urban area since MWFC 2 is located in the city center. It is essential to mention that the river currents go from MWFC baseline to MWFC 2, and between both, it is located a COVID Hospital and a dense urban area, whose wastewater is poured into the Tula River.

The number of SARS-CoV-2 RNA viral copies/L in MWFC baseline was 5.68 × 10^4^, since 7.81 × 10^4^ in MWFC 2. That represents a 37.4% increase in viral copies. The objective of having a baseline in this zone is to visualize the change in the number of viral copies/L before and inside an urban area in the same wastewater body.

### 4.3. Number of Infections Monte Carlo Model

The Monte Carlo model allowed a statistically acceptable estimate distribution of possible cases of people infected with COVID19. Because this model is a non-deterministic method, we report the results as follows: median (minimum value–maximum value). Considering the median, we obtain the following results order from highest to lowest in terms of the number of infected people: MEMHS (S1), MMHS (S1), MMHS (S2), MEMHS (S2), MWFC 3 (S2), MWFC 2 (S1), MEMI (S1) and MNMC (S2), with 13,200, 7864, 2824, 2044, 977, 349, 251 and 189 infected persons, respectively. 

While for the prevalence we have a different order from highest to lowest: MEMHS (S1) with 0.417, MWFC 3 (S2) 0.174, MMHS(S1) 0.075, MEMHS (S2) 0.064, MWFC 2 (S1) 0.034, MMHS (S2) 0.027, MEMI (S1) 0.016, MNMC (S2) 0.014. All these values are detailed in Table 2. 

The prevalence obtained has a significant variation between them; it goes from 1.4% infected population for MNMC (S2) to 41.7% in MEMHS (S1), while official data reports a prevalence of between 0.1% to 0.3% infected population.

Considering the median, the number of infected people in the model goes from 6.5 to 266 times greater than Government reported cases, however we have large variations between the minimum and maximum data obtained by model. The data details of all the sample points are shown in the following Table 3.

## 5. Discussion

### 5.1. Micro-Basin Population and General Viral Waste Determination

Spatial analysis methods were performed in this work in order to approach the amount of population that pours wastewater into the water treatments system, waterway, or river where samples were taken. Specifically, micro watershed delimitation (using digital elevation model) and census demographic data [23] helped estimate this parameter that estimates the number of inhabitants per micro watershed. Other works have used GIS and spatial methods in studies about SARS-CoV-2 in wastewaters but only about spreading the virus and its correlation with atmospheric factors ([24,25]).

We contribute to developing a methodology that offsets the lack of information of developing countries using GIS and comparing the official data. This methodology is proposed for pandemic monitoring through time, especially for early stages and possible outbreaks of different infectious agents. This manuscript is the first model adaptation to Latin American developing countries’ characteristics, hence, contributes a method that permits in a future narrow the values of the Monte Carlo model variables and therefore obtains results with smaller intervals and closer to reality.

Developed countries have performed this Monte Carlo model in similar works ([13,26]). Nevertheless, data in those countries is usually more available, validated, and public. That reach more sophisticated methods, e.g., simulations with several variables about wastewater or ([24,26]). As a developing country, Mexico is still improving systems to allow open and public geo-statistic data and drives digitization processes through geographic information systems. The lack of geographic information about drainage networks is solved in this work by using geoprocessing methods like watershed delimitation and census data, as described before. This perspective could be helpful for countries with a similar lack of data.

Another relevant factor that makes a difference between developing and developed countries is that wastewaters are poured out in rivers or waterways and not only in covered drainage systems that conduct wastewaters into water treatments system. That causes uncertainty of how much people produce the wastewater. For this reason, our study results in a considerable interval.

It is essential to mention that a decrease in RNA copies in the second-day sample (Figure 3) is attributed to increased precipitation in sampling points. That is a good point of reference to have certainty of viral waste determination methodology. Furthermore, it is also remarked in recent review works that dilution can affect final determination ([17,27]). This means a limitation for this study, and is related to the method used for virus concentration. In SARS-CoV-2 surveillance in wastewater analysis, the polyethylene glycol method can give variable final results, for this has some authors suggesting an optimization process for virus concentration [28].

### 5.2. Baseline Comparison in MWFC

Our baseline sample in MWFC was an excellent reference to support the efficiency of viral waste determination. The increase in RNA viral copies is due to the presence of the COVID Hospital and dense urban area wastewater production generated between the baseline point and MWFC 2. We suppose there are many infected persons in COVID Hospitals, increasing in dense urban areas.

### 5.3. Number of Infections Monte Carlo Model

In our Monte Carlo model results, we report a considerable interval. The minimum and the maximum values correspond to the extreme values of Monte Carlo distribution of possible cases of people infected with COVID19, using a 90% of the confidence interval. Although the study has a suitable confidence interval, the interval obtained is significant due to the variables’ uncertainty; some of the intervals used in the variables correspond to other countries’ people. Due to lack of precision bounded local information in “number of inhabitants in the micro-basin”, “feces per capita” and “number of copies of SARS-CoV-2 RNA per gram of stool” variables in Mexico, we have to consider extensive intervals in the model. It is an excellent opportunity area for future research to find more exact values for these variables in the Mexican population. 

The values obtained in this study were high compared to the data reported by ([13]), who detected the viral load of SARS-CoV-2 in concentrations of 19 to 120 copies/L in Australia, where the infrastructure for epidemiological studies is implemented as a monitoring strategy for infectious agents. On the other hand, a review reported by [29] has a compilation of 25 publications related to the determination of SARS-CoV-2 in the water of type: sewage, sewage, and river water, sewage, river, river water, sludge, primary sludge, sewage, sludge, soil and pond, water, surface water simples, and hospital effluent. The methods used to determine the viral RNA content were RT-qPCR, RT PCR, Nested PCR, qPCR, and nested PCR, PCR, and qPCR. The countries where these studies have been carried out were Australia, the USA, Brazil, Spain, France, Italy, Germany, Switzerland, the Netherlands, India, Turkey, Israel, Japan, Ecuador, Saudi Arabia, South Africa, and China. In general, it can be said that the average minimum value of viral copies per liter is 1.01 × 10^5^ with a standard deviation of 4.00 × 10^5^. In contrast, the average maximum value is 2.44 × 10^7^ with a standard deviation of 1.05 × 108, where the value depends on the sampling method, the concentration of the sample, and the physicochemical properties of the water. However, under this context, the samples evaluated here are within the minimum and maximum average interval reported by [29].

### 5.4. Difference between Monte Carlo Model Results and Officially Reported Cases

In all the area samples, we report a large number of infected people in comparison with the Hidalgo State Government; this is mainly because of the lack of uncertainly in variables used in the Monte Carlo model and the low COVID-19 testing level in Mexico (its maximum value is 0.4 tests daily per thousand people, while France or the USA had 10.17 and 6.8 tests daily per thousand people [30]). The latter is due to the SARS CoV-2 pandemic response corresponding to national policy focused on social distancing and isolation measures. 

The suitable variables to improve accuracy at a regional level are as follows: (a) wastewater per capita, (b) feces per capita, and (c) number of copies of SARS-CoV-2 R.N.A. per gram of stool (copies/g). By narrowing data values on variables, we can delimitate the model results to smaller and more accurate intervals.

We are sure that an increase in the bounded variables data used in the model would get our results closer to reality. However, these data cannot be very close to officially reported cases unless the level of response from Government testing increases.

### 5.5. Prevalence Monitoring

Although our model presents results with a large interval, we propose it as a pandemic monitoring method that complements Government data. At the same time, this model can help in early RNA detection of infectious agents in water bodies, allowing a prompt response to other viral diseases. Some interesting considerations about prevalence estimation uncertainties are described in a five steps approach (virus shedding; in-sewer transportation; sampling and storage; analysis of SARS-CoV-2 RNA concentration in wastewater; back-estimation) and were considered in this work [31]. 

Finally, agriculture is one of the most important economic activities in the west of the State of Hidalgo, in a widely studied Mezquital Valley (MWFC sample) [32] where food production yields the south strip of Hidalgo State and the north area of Mexico City. In this valley, where the primary water source for irrigation is wastewater, it is essential to have more information about the infectiousness of SARS CoV-2.

In this regard, ([27]) reported the virus’s genetic material in WWTP both in the primary effluent and in the treated secondary effluent. However, when the tertiary treatment processes were present in the treatment train, no genetic material was detected in the effluent stream. It has been reported that other enveloped viruses are not eliminated by conventional primary ([33,34]) and secondary wastewater treatment [35]. As a result, they can be released into natural waters and bioaccumulate inside aquatic species such as shellfish [36]. Humans, in turn, can become infected by viruses in natural waters by drinking contaminated water or eating contaminated food, as well as by bathing or inhaling bioaerosols from contaminated waters [35]. The decrease in viral load in aquatic environments depends on the time elapsed since its release, the viral resistance to natural and artificial disinfection factors, and dilution. Unlike most enteric viruses usually found in wastewater, coronaviruses are enveloped and considered less resistant in the environment [37]. Currently, no studies have explored the infectivity of SARS-CoV-2 in wastewater treatment plants as a function of time, and there are no estimates available on the minimum infectious dose of SARS-CoV-2 in wastewater required to cause infection in humans ([27,38]).

In the Mezquital Valley, the use of wastewater without treatment could represent a risk to health in general and under the context of the SARS-CoV-2 pandemic. On the other hand, the population that lives near the receiving bodies of wastewater, workers in wastewater treatment plants, and workers in the health sector could be at risk because the virus is transmitted through the formation of aerosols or fecal-oral route. Inhalation of viral aerosols and exposure to contaminated waste and sludge can potentially produce infections. Hence, extra cautions must be taken to minimize the generation of aerosols during wastewater treatment and the handling of sewage sludge. Workers at hospitals, quarantine facilities, and WWTPs should be provided with appropriate personal protective equipment (PPE). Health and WWTP workers should follow the WHO and WASH guidelines to minimize the spread of this virus [39].

## 6. Conclusions

In the mid-2020s, Mexico experienced its highest level of contagion to date; however, the level of diagnosis had its limitations because the national policy was focused on social distancing and containment of mobility rather than testing; this led to a high level of under-diagnosis. 

The work proposed here represents an alternative to identify the level of under-diagnosis by calculating prevalence and thus reducing the uncertainty of the level of contagion. Given the lack of information on drainage and sewerage, the data obtained through census information and delimitation of micro-basins represented a practical and consistent solution since it was based on local information.

Viral RNA residues (0–197,655 copies/L) were determined in wastewater from the five central municipalities in Hidalgo State. The Monte Carlo model shows many SARS CoV-2 infected persons, compared to official reports of the five municipalities, we obtained a percentage of the infected population from 1.4% to 41.7%, while the official data reports 0.1–0.3%, which tell us that it is needed to consider variables better and improve national policies regarding pandemic monitoring response. 

Monte Carlo is sturdy and could be adapted to locally generated data, yielding consistent and higher results than those officially reported. It is the first work adapted to the characteristics of Latin American developing countries that do not have sufficient information and infrastructure in the wastewater collection system.

We showed that precipitation affects the detection levels of viral copies due to dissolution, as was observed in the second sampling campaign in which there were higher precipitation levels. However, it should be considered that the method has a sufficient level of sensitivity to detect dilute concentrations of viral RNA. 

Hidalgo is about to experience the third wave of infections. These techniques can be beneficial for early monitoring of highly populated areas and sanitary fences; this allows the establishment of public policies such as mobility reduction, sanitary fences, social distancing measures, and the improvement of water sanitation infrastructure.

## Figures and Tables

**Table 1:** COVID-19 cases in municipalities where the sampling sites are located.

Municipality	Confirmed Infected People Reported by the Government at Sampling Dates ^1^	Sampling Date ^2^	Municipality Place in Hidalgo State According to the Number of Confirmed Infected People Reported by the Government ^1^	Municipality Population in 2020
MEMHS	319	June 30th (S1)	2nd place	187,701
	347	July 7th (S2)		
MMHS	848	June 30th (S1)	1st place	281,702
	959	July 7th (S2)		
MWFC ^2^	124	June 30th (S1)	9th place	117,083
	142	July 7th (S2)		
MEMI	158	June 30th (S1)	6th place	55,061
	177	July 7th (S2)		
MNMC	283	June 30th (S1)	3rd place	140,727
	321	July 7th (S2)		

^1^ Data reported by the health sector agencies in collaboration with the Federal Government on the website: https://coronavirus.hidalgo.gob.mx/ [18] accessed on 7 July 2021. June 2nd 30th sampling is represented by S1, while July 7th sampling is represented by S2. ^2^ MWFC include: MWFC, MWFC 2 and MWFC 3.

**Table 2:** Estimate of cases of infected people and prevalence based on the Monte Carlo model.

Sample Name	RNA Copies/L	Inhabitants Infected in the Micro-Basin ^1^	Inhabitants Infected in the Municipality ^2^	Prevalence of Infection (%) Median (90%CI)
MEMHS (S1)	197,655	13,200 (3016–62,125)	78,379 (17,909–368,888)	0.417 (0.095–1.965)
MEMHS (S2)	29,885	2044 (506–9843)	12,137 (3005–66,365)	0.064 (0.016–0.311)
MMHS (S1)	32,909	7864 (2997–63,846)	21,182 (8073–184,227)	0.075 (0.028–0.61)
MMHS (S2)	11,762	2824 (700–13,098)	7607 (1885–37,794)	0.027 (0.006–0.125)
MWFC 2 (S1)	78,174	349 (209–6658)	4076 (2441–106,647)	0.034 (0.020–0.664)
MWFC 3 (S2)	8159	977 (17–604)	20,438 (356–20,392)	0.174 (0.003–0.107)
MEMI (S1)	30,230	251 (215–4553)	917 (785–18,835)	0.016 (0.014–0.302)
MEMI (S2)	Not detected	-	-	-
MNMC (S1)	Not detected	-	-	-
MNMC (S2)	9234	189 (29–887)	2055 (315–14,589)	0.014 (0.002–0.068)

^1^ Theoretical data obtained with Monte Carlo model: median (minimum value–maximum value). ^2^ Extrapolation with the supposition that exists the same number of infected people by km^2^ than micro-basin sample site.

**Table 3:** Times greater of infected people reported in the model in comparison with official reported cases.

Sample Name	Hidalgo Government Reported Cases	Ratio ^1^
MEMHS (S1)	319	245.7 (56.1–1156.3)
MEMHS (S2)	347	39.7(9.8–191.2)
MMHS (S1)	848	26.7 (10.1–217.2)
MMHS(S2)	959	8.4 (2.1–39.4)
MWFC 2 (S1)	124	45(26.9–860)
MWFC 3 (S2)	124	266 (4.6–164.4)
MEMI (S1)	158	6.5(5.6–119.2)
MEMI (S2)	177	-
MNMC (S1)	283	-
MNMC (S2)	321	9.6 (1.4–45.4)

^1^ This data is the result of the next ratio: number of infected people in municipality obtained by model/Hidalgo official reported cases: median (minimum value–maximum value).

## Data Availability

Data are available in a public repository. This data can be found here: https://github.com/luzhflores/sars_cov_2_mex. This data was upload on 2 may 2021.
